# Investigation of Amphibian Mortality Events in Wildlife Reveals an On-Going Ranavirus Epidemic in the North of the Netherlands

**DOI:** 10.1371/journal.pone.0157473

**Published:** 2016-06-17

**Authors:** Jolianne M. Rijks, Bernardo Saucedo, Annemarieke Spitzen-van der Sluijs, Gavin S. Wilkie, Alphons J. A. M. van Asten, Jan van den Broek, Roschong Boonyarittichaikij, Marisca Stege, Fleur van der Sterren, An Martel, Frank Pasmans, Joseph Hughes, Andrea Gröne, Steven J. van Beurden, Marja J. L. Kik

**Affiliations:** 1 Dutch Wildlife Health Centre, Utrecht University, Utrecht, The Netherlands; 2 Department of Pathobiology, Utrecht University, Utrecht, The Netherlands; 3 Reptile, Amphibian and Fish Conservation Netherlands, Nijmegen, The Netherlands; 4 MRC-University of Glasgow Centre for Virus Research, Glasgow, United Kingdom; 5 Department of Farm Animal Health, Utrecht University, Utrecht, The Netherlands; 6 Department of Pathology, Bacteriology and Avian Diseases, Ghent University, Merelbeke, Belgium; CSIRO, AUSTRALIA

## Abstract

In the four years following the first detection of ranavirus (genus *Ranavirus*, family *Iridoviridae*) infection in Dutch wildlife in 2010, amphibian mortality events were investigated nationwide to detect, characterize and map ranaviruses in amphibians over time, and to establish the affected host species and the clinico-pathological presentation of the disease in these hosts. The ultimate goal was to obtain more insight into ranavirus disease emergence and ecological risk. In total 155 dead amphibians from 52 sites were submitted between 2011 and 2014, and examined using histopathology, immunohistochemistry, virus isolation and molecular genetic characterization. Ranavirus-associated amphibian mortality events occurred at 18 sites (35%), initially only in proximity of the 2010 index site. Specimens belonging to approximately half of the native amphibian species were infected, including the threatened *Pelobates fuscus* (spadefoot toad). Clustered massive outbreaks involving dead adult specimens and ranavirus genomic identity indicated that one common midwife toad virus (CMTV)-like ranavirus strain is emerging in provinces in the north of the Netherlands. Modelling based on the spatiotemporal pattern of spread showed a high probability that this emerging virus will continue to be detected at new sites (the discrete reproductive power of this outbreak is 0.35). Phylogenetically distinct CMTV-like ranaviruses were found in the south of the Netherlands more recently. In addition to showing that CMTV-like ranaviruses threaten wild amphibian populations not only in Spain but also in the Netherlands, the current spread and risk of establishment reiterate that understanding the underlying causes of CMTV-like ranavirus emergence requires international attention.

## Introduction

The long-term effects of ranavirus disease on amphibian communities and eco-systems are a matter of concern worldwide [1–2]. In the Netherlands, ranavirus disease was detected for the first time in wildlife in 2010, when amphibians died in high numbers in a pond of the Dwingelderveld National Park (DNP) [3]. Partial genetic characterization showed that the outbreak was caused by a common midwife toad virus (CMTV)-like ranavirus (genus *Ranavirus*, family *Iridoviridae*) [3]. CMTV was first detected in Spain, where it was shown to pose a threat for wild amphibian populations [4–6].

Despite being a disease notifiable to the World Organization of Animal Health [7], reported ranavirus infections in wild amphibians in continental Europe are scarce and local. Aside from the Netherlands and the Iberian Peninsula, ranaviruses have been detected in wild amphibians in Belgium [8], Croatia [9], Denmark [10], France [11], Germany [12], and in wild amphibians taken into captivity in Italy [13]. Ranaviruses characterized as CMTV-like based on PCR and sequencing of the partial major capsid protein (MCP) gene, were detected in clinically healthy larvae of an exotic species *Lithobates catesbeianus* (American bullfrog) in Northern Belgium [8] and in *Rana temporaria* (common frog) in Southeastern France (Mercantour National Park) [11]. In Denmark [10], Germany [12] and Italy [13], the partially characterized ranaviruses also clustered closely with CMTV [12, 14] and were often associated with mortality events involving *Pelophylax* spp. (water frogs) [9–10, 12–13]. There is a need for more long-term multidisciplinary studies that assess how ranaviruses affect sympatric amphibian populations over time in Europe.

In the four years (2011–2014) following the DNP die-off, we investigated amphibian mortality events nationwide to detect, characterize and map the distribution of ranaviruses in amphibians over time, and to establish the affected host species and the clinico-pathological presentation of the disease in these hosts. The underlying assumption was that spatiotemporal patterns of ranavirus associated mortality events, in combination with molecular characterization of the virus, and with disease patterns in hosts, would provide insight into emergence and contribute to defining ecological risk [2]. The study allowed us to detect ranaviruses, document their effects, identify an emerging virus and determine the probability of spread to new sites.

## Materials and Methods

### Detection

From 2011 onwards members of the foundation ‘Reptile, Amphibian, and Fish Conservation Netherlands’ (http://www.ravon.nl) and the public were requested to submit dead specimens from amphibian mortality events for post-mortem examination at the Dutch Wildlife Health Centre (under permit no. FF/75A/2008/075). Only specimens that were found dead were accepted, therefore no permission of the Committee on the Ethics of Animal Experiment was required. Submissions with at least one non-autolytic dead specimen were included in this study.

Ranavirus screening involved histological examination and DNA extraction, PCR and sequencing of the partial major capsid protein (MCP) gene of organ material as previously described [3, 15]. When results were positive, immunohistochemistry was performed on organ tissue of at least one specimen per site to confirm infection. A polyclonal rabbit anti-European catfish virus antibody (kindly donated by G. Bovo, Instituto Zooprofilattico Sperimentale delle Venezie, Italy) was used as a primary antibody. The protocol was based on a published method [16] and slightly modified, as detailed in (S1 Text). The sites from which the specimens were obtained were numbered chronologically and mapped per year for spatiotemporal analysis (Table 1). DNP is referred to as the index site (no.0).

**Table 1:** Geographical coordinates of sites (WGS 84), with longitude in decimal degrees East (x), and latitude in decimal degrees North (y).

Site no.	x	y	Year	Site no.	x	y	Year	Site no.	x	y	Year
0	6.38	52.78	2010	18	5.06	51.64	2012	36	6.31	52.95	2013
1	6.22	52.98	2011	19	5.95	52.30	2012	37	6.29	52.94	2013
2	5.08	51.67	2011	20	4.59	52.44	2012	38	6.21	52.85	2013
3	7.03	52.68	2011	21	4.49	52.17	2012	39	5.99	50.78	2013
4	5.44	51.56	2011	22	5.83	51.96	2012	40	6.23	52.00	2014
5	6.08	51.33	2011	23	5.78	52.68	2012	41	5.94	52.31	2014
6	5.62	51.88	2011	24	6.11	51.17	2012	42	5.21	52.13	2014
7	6.31	52.65	2011	25	6.28	52.63	2012	43	5.85	52.41	2014
8	5.80	52.38	2011	26	4.49	52.15	2012	44	5.98	50.93	2014
9	6.53	52.80	2011	27	5.92	50.80	2012	45	6.32	52.70	2014
10	4.69	52.60	2011	28	6.06	53.09	2012	46	6.11	52.96	2014
11	6.47	52.75	2011	29	5.82	51.47	2012	47	6.04	51.17	2014
12	4.76	52.50	2011	30	5.93	52.34	2013	48	6.38	52.97	2014
13	6.21	52.77	2011	31	5.22	52.09	2013	49	5.85	51.04	2014
14	6.30	52.00	2011	32	5.95	52.06	2013	50	6.11	51.17	2014
15	5.45	51.43	2011	33	5.02	51.63	2013	51	6.37	52.73	2014
16	5.33	51.59	2011	34	4.49	52.15	2013	52	5.85	52.41	2014
17	5.80	51.55	2012	35	6.21	52.86	2013				

### Virus genetic characterization

Ranavirus genetic characterization involved sequencing of the complete genome for one isolate [17] and six additional partial genes for other ranavirus samples [6]. The methods used to obtain and sequence the full genome are detailed elsewhere [17]. To reconstruct the phylogeny of the fully sequenced isolate, common_midwife_toad_ranavirus_NL_KP056312, twenty-six core protein sequences from 17 full genomes from members of the family *Iridoviridae* were extracted from Genbank, details of which can be found in (S1 Table). These were aligned with the isolate’s 26 core iridovirus proteins using MAFFT version 7 [18] to produce a protein alignment for each gene. The core set of genes was concatenated and the best protein substitution models for each gene partition were selected using PartitionFinder [19]. The maximum likelihood phylogeny was reconstructed using 1000 bootstrap replicates using RAxML (version 8) [20].

For the partial characterization, DNA fragments were amplified by PCR, ligated into pGEM-T-Easy vector (Promega Co., Madison), cloned into competent *E*. *coli* (strain HB 101) and sequenced from both ends by Sanger sequencing (Macrogen Europe, Amsterdam). To reconstruct the phylogeny of the partially sequenced ranaviruses, 7 partial gene sequences previously used to characterize CMTV-like ranaviruses [6] were determined for 16 other ranaviruses from the Netherlands. Partial gene sequences were manually concatenated and aligned with the corresponding partial gene sequences of common_midwife_toad_ranavirus_NL_KP056312 and of 21 related iridoviruses extracted from Genbank using Clustal Omega. The details of the partially sequenced viruses can be found in (S2 Table). The tree was constructed by using the best-fit model (General Time Reversible + Gamma distribution (GTR+G) model) in MEGA 6.06. Maximum likelihood phylogeny was reconstructed using 1000 bootstrap replicates.

### Disease characterization

Three main lesions previously associated with ranavirus infection in the Netherlands were scored in selected tissues to compare presence and severity of lesions between groups of specimens. These lesions were the number of pale basophilic intra-cytoplasmic inclusion bodies (ICIB; scores: 0 if none seen; 1 if < 5 per high power field; 2 if 5–10 per high power field; 3 if > 10 per high power field), the extent of necrosis (scores: 0 if none seen; 1 if involving < 5% of organ; 2 if involving 5%-50% of organ; 3 if involving >50% of organ), and the level of vascular damage (scores: 0 if none seen; 1 if mild; 2 if moderate; 3 if severe). Each lesion was first scored in hematoxylin-and-eosin stained liver, kidney, spleen, intestine and skin tissue as present. All slides were evaluated separately by two veterinary pathologists and when discrepancies were observed, both parties met to reach a definitive consensus. The scores obtained for the slides were then averaged per individual specimen (average of the different tissue scores). These average scores were used in the statistical analyses that were performed to assess how revealing the lesions were for the presence of ranavirus infected specimens at a site (Chi-square tests), and for assessing species differences in lesion severity (ANOVA).

The life stages that died at the confirmed sites were compared to data from phenological frequency diagrams. These species specific diagrams are based on decades of monitoring data collected and analyzed by RAVON for amphibians in the Netherlands. They use the frequency of observation of a life stage on a given day of year, providing a proxy of numbers and activity rates of life stages at different times of the year [21]. The resulting bar diagrams indicate, per species and life stage, the relative proportion of sightings occurring during 15 days (24 time periods). If the outbreak was detected during the time period with the highest proportion of sightings, the phenological frequency of the life stage was considered “high”. If the outbreak was detected during a time period that proportion of sightings was less than five percent of the total sightings, the phenological frequency of the life stage was considered “low”. In between, it was called “moderate”.

Submitters of specimens were asked to estimate the number of dead animals during the initial event, and provide their observations in regards to amphibian population trends at the site if ranavirus presence was confirmed. Specifically, they were asked to record the number of dead and live specimens per species and life stage, indicating whether these numbers were counted or estimated, and keep track in the following years. No fixed dates were given for recording these findings. All submitters were contacted in September 2014 for a final overview.

### Modelling the outbreak

When a ranavirus disease outbreak is detected, it is relevant to understand how likely it is that the outbreak will continue, taking into consideration that measures may be taken to prevent human-mediated disease spread to new sites. This was statistically quantified by calculating the discrete reproductive power of the identified outbreak using a nonhomogeneous birth process, with “site” as the epidemiological unit. The nonhomogeneous birth process takes into account the fact that this study did not identify sites with susceptible specimens, but rather sites with infected specimens, and possibly with a certain delay [22]. The calculated discrete reproductive power is the probability that a detected infected site reproduces, i.e., that the infection spreads from the site to susceptible populations at other sites. For the statistical model, probability distributions from the Burr family were used, as detailed elsewhere [22–24].

The data set used in the calculation of the reproductive power consisted of the sequence of the number of sites per year where confirmed ranavirus-associated mortality events occurred and were detected (i.e., the number of new sites), for the period and area under investigation. The time-interval “year” considers the seasonality of ranavirus disease and of amphibian activity. Given the limited number of infected sites detected per year, the reproductive power was considered constant.

In order to calculate a confidence interval for the reproductive power, a Monte Carlo procedure (1000 runs) was used with the estimated distribution of the birth process, which is a negative binomial distribution. If after the initial case no more infections occur, the reproductive power is zero (0); the probability that there is no reproduction is 1-reproductive power. That this happens four years in a row (2011–2015) following the initial case (2010) has a probability of (1-reproductive power)^4. A confidence interval for the discrete reproductive power estimate can be calculated considering only those cases (runs) where after the initial site at least one other site was infected, which happens in about (1-[1-reproductive power]^4) of the cases. Calculations were made using R version 3.0.3 [25]. Script details are found in (S2 Text).

## Results

### Multiple sites with ranavirus-associated amphibian mortality

**Fig 1:**
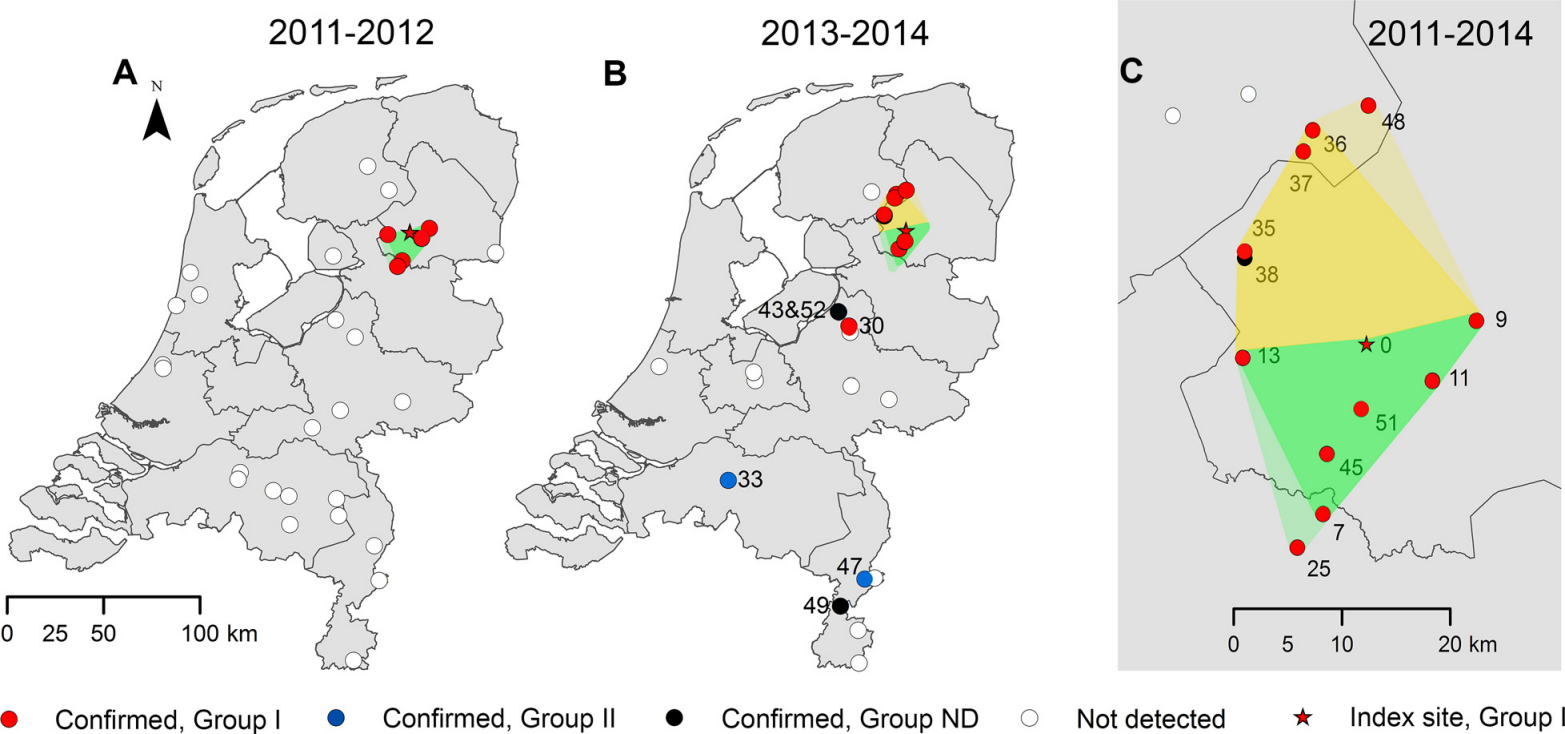
Spatiotemporal distribution of ranavirus associated amphibian mortality events, the Netherlands, 2011–2014. (A) Country overview, 2011–2012. (B) Country overview, 2013–2014. (C) Close-up of area around index site, 2011–2014. The green surface contains all sites with confirmed ranavirus-associated mortality events in 2011 (dark green shade), extending southwards in 2012 (light green shade). The yellow surface is the area to the north of the index site where additional events occurred ≤ 20 km from the index site in 2013 (dark shade) and 2014 (light shade). The numbers correspond to the site numbers for sites with confirmed ranavirus presence. ND = Not determined.

**Fig 2:**
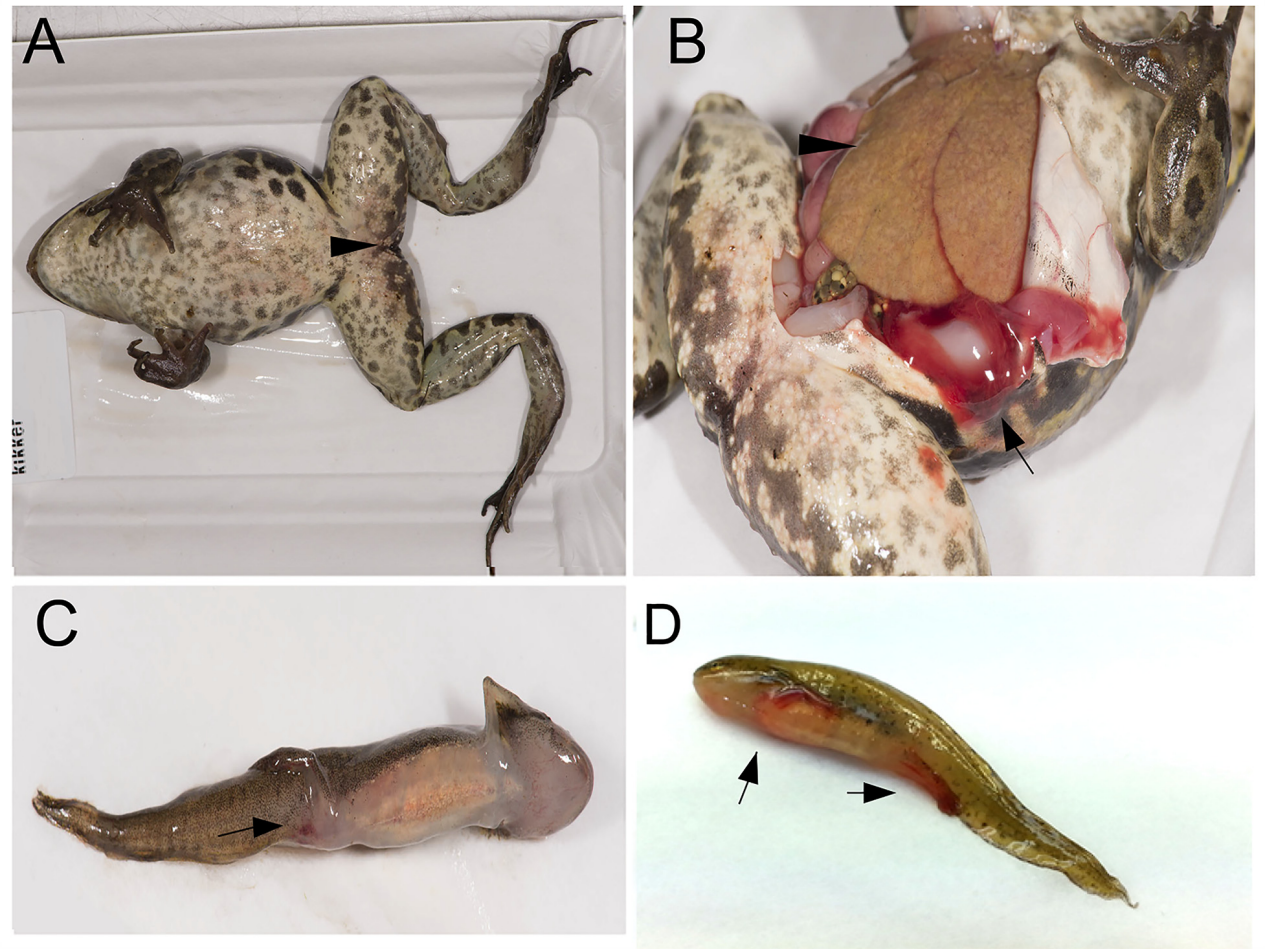
Macroscopic lesions in amphibians naturally infected with CMTV-like ranavirus from the Netherlands. (A) Adult *Pelophylax* kl. *esculentus* with mild erythema of the skin from the inguinal region (black arrow head). (B) Internal inspection of animal from Fig 2A with enlarged pale liver showing marked hepatic necrosis (black arrow head) and areas of hemorrhage in the coelomic cavity (black arrow). (C) Neotenic adult *Lissotriton vulgaris* presenting with focal area of hemorrhage in the cloaca (black arrow). (D) Smooth newt larvae presenting with hemorrhages in the limbs (black arrows).

**Fig 3:**
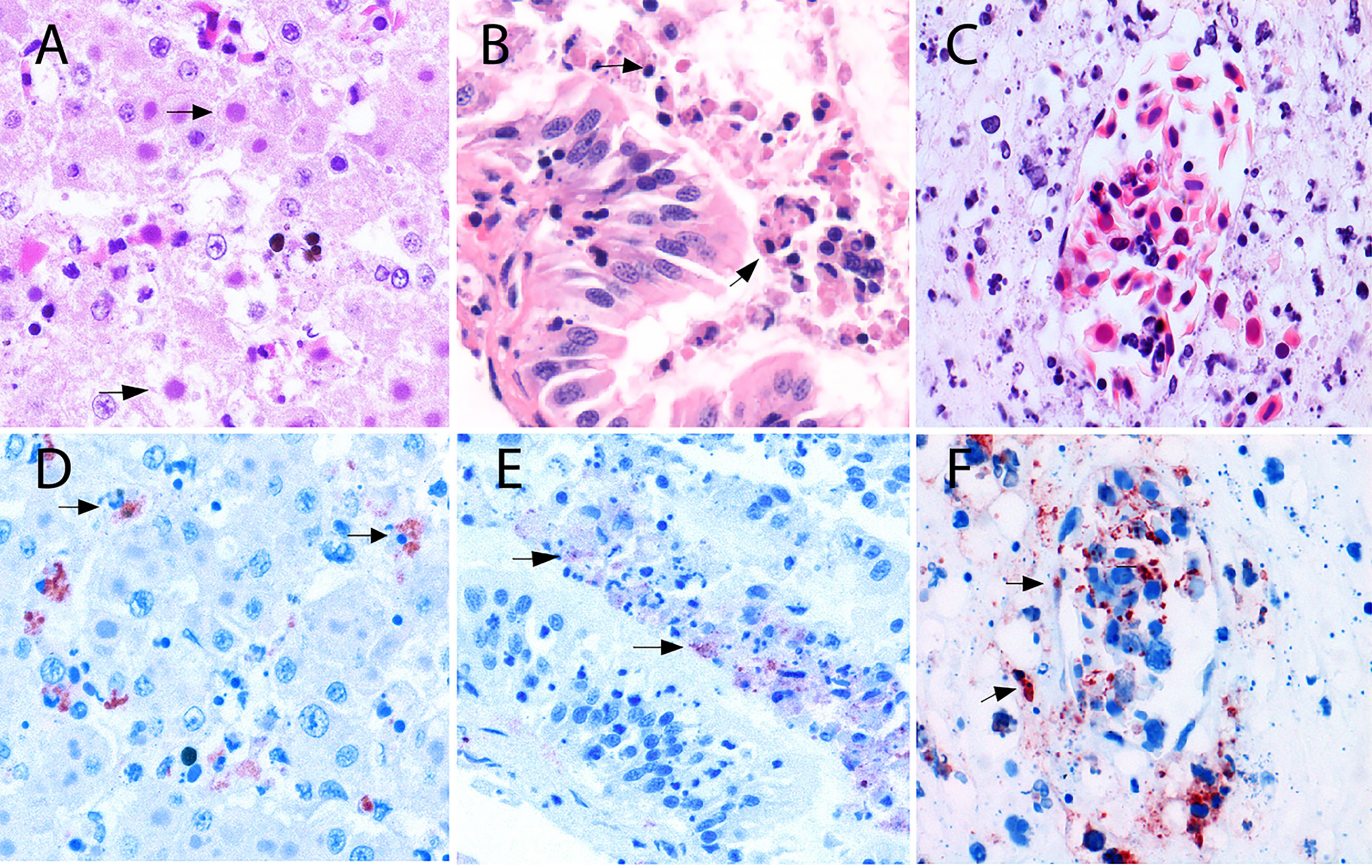
Microscopic lesions in amphibians naturally infected with CMTV-like ranavirus from the Netherlands. These lesions illustrate the criteria considered in the double-blind semi-quantitative scoring system. (A) Hematoxylin and eosin (H&E)-stained liver of a *Pelophylax* kl.*esculentus* infected with CMTV-like ranavirus, black arrows indicate basophilic intracytoplasmic inclusion bodies in the hepatocytes. Original magnification ×400. (B) H&E–stained intestine of a *Pelophylax* kl.*esculentus* infected with CMTV-like ranavirus presenting with detachment and necrosis of enterocytes in the apical portion of the mucosal villi (black arrows). Original magnification ×400. (C) H&E-stained section of the intestinal submucosa with evident vascular damage characterized by perivascular edema and collections of karyorrhectic cell debris. Original magnification ×400. (D) Immunohistochemistry of a serial section from figure A using an anti-European catfish virus (ECV) polyclonal antibody, positive immunolabeling is observed in the cytoplasm of affected hepatocytes (black arrows). Original magnification ×400. (E) Immunohistochemistry of a serial section from figure B using ECV polyclonal antibody, positive immunolabeling is observed in numerous necrotic enterocytes exfoliated into the lumen (black arrows). Original magnification ×400. (F) Immunohistochemistry of a serial section from figure C using ECV polyclonal antibody, positive immunolabeling is present in the endothelial cell wall and in the cells scattered throughout the damaged submucosa (black arrows). Original magnification x400.

The investigation of 155 dead amphibians submitted from 52 sites in 2011–2014 confirmed that ranavirus-associated mortality had occurred at 18 sites (35%; Fig 1). The probability of ranavirus detection was not significantly different between submissions of just one animal and submissions of multiple animals (Fisher exact test, one-tailed p = 0.07). The majority of the specimens submitted from the 18 sites (60/69, 87%) tested positive in the partial MCP gene PCR-test. Lesions consistent with ranavirus infection as described previously [1, 3, 4] were observed in specimens from 16/18 sites where ranavirus was detected by the PCR-test, and infection was confirmed by immunohistochemistry at 17/18 sites (Figs 2 and 3). No other major pathogens were identified in specimens from amphibian mortality events associated with ranavirus. Details are found in (S3 Text).

Initially, in 2011 and 2012, the ranavirus-associated mortality events clustered around the index site DNP. Subsequently, they were detected more frequently (13/23 events in 2013–2014 versus 5/29 events in 2011–2012; Yates *X*^2^ = 7.095; p = 0.008) and over a larger area, occurring at 55–60 km and > 150 km from DNP, as well as still within 20 km but more northwards (Fig 1; Table 2). All events occurred in the warmer months of the year (April–September, average temperatures around 10°C or higher), as reported elsewhere [26].

**Table 2:** Characteristics of ranavirus associated mortality events, grouped by distance from the index site (no. 0). ND = Not determined. NA = Not applicable. All = No, or virtually no, live specimens remain in the water body in the immediate aftermath of the initial mortality event.

Km to index site	Phylo- group no.	Site no.	Site type*	Month and year identified	(Estimate of) numbers dead	Life stages†-species‡ affected, per phenological frequency of the life stage at time of the event			Situation *Pelophylax* spp. in 2014
						High	Moderate	Low	
≤20	I	7	G	May 2011	100–1000 (all)	L-*Bb*	A-*P*, A-*Bb*		Unknown
	I	9	G	Jul. 2011	100–1000 (all)	L-*Lv*	L- *P*	A-*P*	Pond renovated
	I	11	G	Aug. 2011	10–100 (all)	J-*Lv*	L- *P*, L- *Lv*, J- *P*	A-*P*	Reduced §
	I	13	G	Sep. 2011	10–100			A-*P*	Reduced §
	I	25	La	Jun. 2012	≥1000	L-*Pf*	L- *Lv*		Unknown
	I	35	G	Aug. 2013	100–1000		L- *P*	A-*P*	Reduced §
	I	36	La	Aug. 2013	≥1000 (all)	J-*P*, J-*Lv*	L- *P*	A-*P*	Reduced §
	I	37	N	Aug. 2013	100–1000 (all)	J-*P*, J-*Lv*	L- *P*	A-*P*, A-*Lv*	Reduced §
	ND	38	G	Sep. 2013	10–100 (all)			A-*P*, A-*Lv*	Reduced §
	I	45	G	May 2014	10–100		A-*P*		NA
	I	48	N	Jul. 2014	10–100	L-*P*, L-*Lv*	A-*P*	A-*Lv*	NA
	I	51	N	Sep. 2014	10–100			A-*P*	NA
55–60	I	30	S	Apr. 2013	10–100		A-*Rt*		Unknown
	ND	43¶	N	May 2014	1–10	A-*Lv*			NA
	ND	52¶	Po	Sep. 2014	1–10			A-*Bb*	NA
>150	II	33	N	Jul. 2013	10–100	L-*P*, L-*Lv*	A-*P*		No effect
	II	47	La	Jul. 2014	10–100		L-*Pf*#, A-*P*		NA
	ND	49¶	La	Aug. 2014	1–10		L-*P*		NA

* G = garden pond; La = landscaped pool (natural pool remodeled by humans); N = natural pool; Po = pond; S = stream.

† L = larvae; J = juveniles; A = (sub-)adults.

‡ *Bb* = *Bufo bufo*; *Lv* = *Lissotriton vulgaris*; *Pf* = *Pelobates fuscus*; *P* = *Pelophylax* spp. (three species *P*. kl. *esculentus*, *P*. *lessonae* and *P*. *ridibundus*, grouped here because visually undistinguishable at larval stage; adult specimens of all three species shown to be infected); *Rt* = *Rana temporaria*.

§ The number of adult *Pelophylax* spp. seen by garden pond owners was ≤ 10% of the pre-epidemic numbers after a year (no. 11, 13, 38) and ≤ 20% of the pre-epidemic numbers after 3 years (no. 11, 13). Egg masses and larvae were often absent. A field visit to sites no. 36 and 37 a year later showed ≤ 10% of the pre-epidemic numbers. Only descriptive data was provided for site no. 35.

¶ At site no. 43, the PCR-test positive specimen (*Lissotriton vulgaris*) was IHC negative, and at sites no. 49 (*Pelophylax* kl. *esculentus*) and no. 52 (*Bufo bufo*), the specimens had no histological lesions consistent with ranavirus. At all other sites, all three methods gave results consistent with ranavirus infection.

# Site no. 47 is a landscaped pool, in which nearly full-grown spadefoot toad larvae were reintroduced in 2014. Therefore, phenological frequency does not really apply to these spadefoot toad larvae. The larvae had hatched and had been raised in captivity from egg masses taken earlier that year from site no. 25, a known ranavirus-positive site.

### Distinct common midwife toad-like ranaviruses in the Netherlands

**Fig 4:**
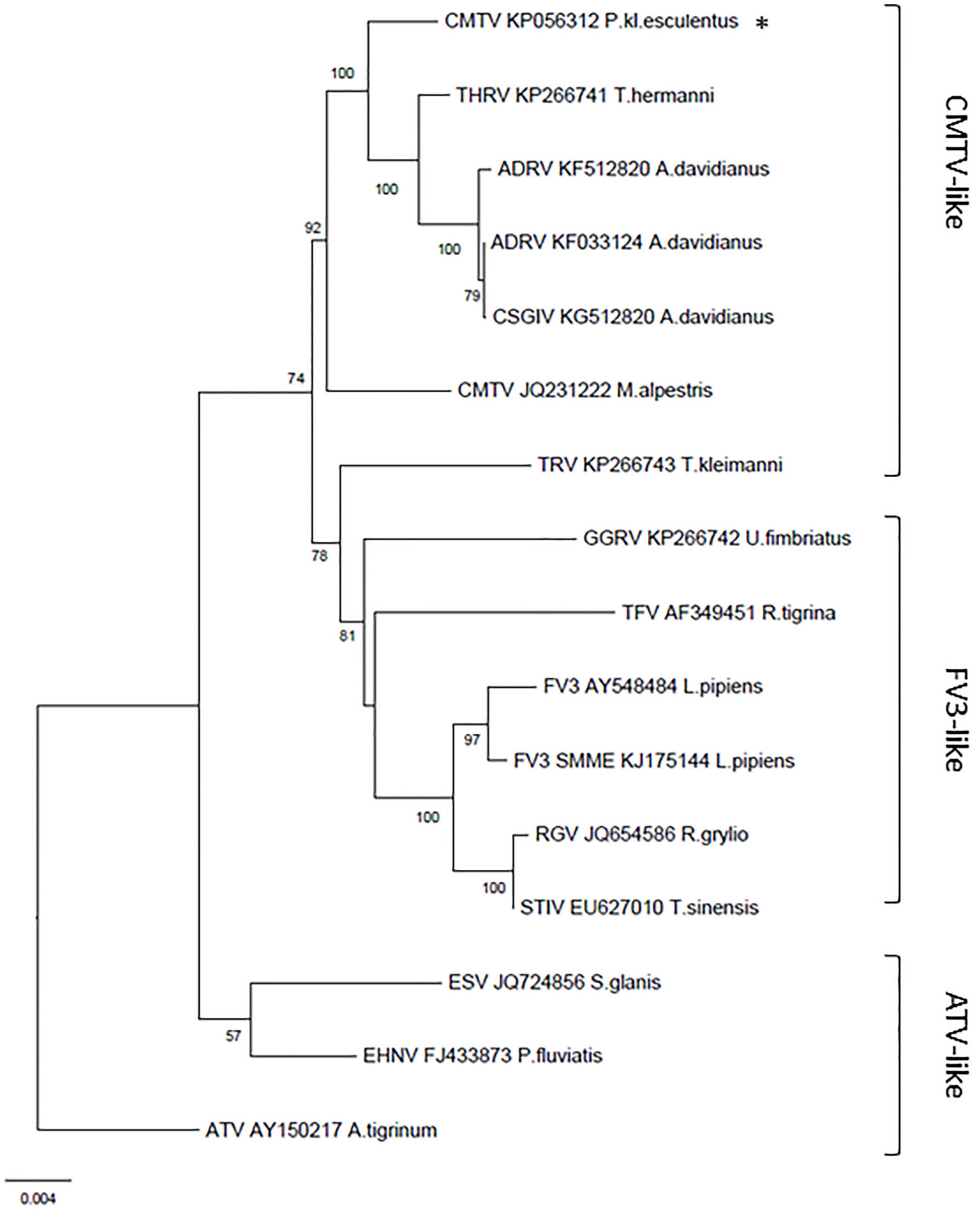
Phylogeny of the fully sequenced ranavirus isolate associated with amphibian mortality at site no. 35. Maximum-likelihood phylogeny based on the 26 iridovirus core proteins of the fully sequenced ranavirus from site no. 35 and other publically available ranavirus genomes. The grouper iridoviruses were used as an outgroup (not shown). The bootstrap support is shown at the nodes.

**Fig 5:**
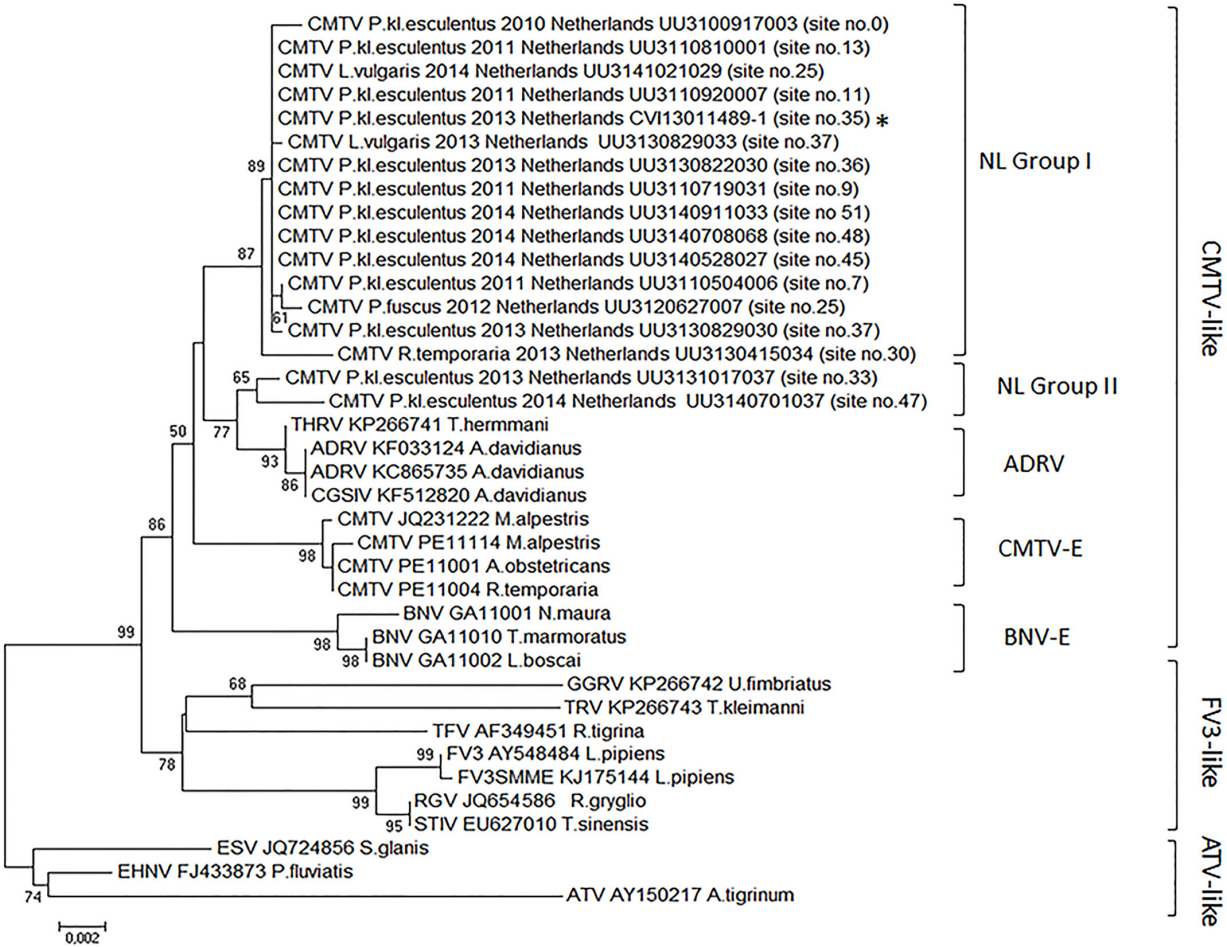
Phylogeny of the partially sequenced ranaviruses associated with amphibian mortality events in the Dutch wildlife. Maximum-likelihood phylogeny of ranaviruses based on concatenated alignments of seven partial gene sequences. The ranavirus samples from the Netherlands cluster in two distinct phylogenetically related groups (NL group I and NL group II) within the CMTV-like ranavirus group, clearly distinct from CMTV from Spain (CMTV-E) and Bosca’s newt virus from Spain (BNV-E). The fully sequenced ranavirus from site no. 35 is indicated with an asterisk (*). The bootstrap support is shown at the nodes. Only values >50% are shown.

Molecular characterization identified two groups of phylogenetically related common midwife toad virus (CMTV)-like ranaviruses [5–6, 17]. At all sites, the partial MCP gene sequences were 99.35–100% identical to CMTV (GenBank accession number JQ231222.1; [5]) and 99.56–99.78% identical to *Andrias davidianus* ranavirus (ADRV; GenBank accession number KC865735.1 [27]), as detailed in (S3 Table). Full genome analysis of a ranavirus isolate from site no. 35 (CMTV_*P*.kl.*esculentus*_2013_Netherlands_isolate_CVI13011489-1) [17] positioned it intermediately between CMTV from Spain [5] and ADRV from China [27] (Fig 4). Characterization of ranaviruses from 16 specimens at 14 other sites using 7 genes showed the presence of 2 groups, phylogenetically distinct from CMTV and Bosca’s newt virus (BNV) found in Spain [6] (Figs 1 and 5). One group (I) contains the ranaviruses detected at the index site DNP and at sites within 20 km of DNP, as well as an additional ranavirus located within 60 km; the other group (II) contains ranaviruses detected more than 150 km from DNP (no. 33 and 47) and ADRV found in captive animals [27–29]. The GenBank accession numbers of the Dutch ranaviruses are available in (S4 Table).

### Clinico-pathological differences observed among species and sites

The specimens infected with ranavirus belonged to 7 of the 16 native amphibian species (Table 2). The *Bufo bufo* (common toad), *Lissotriton vulgaris* (the smooth newt), three *Pelophylax* spp. (waterfrog species; *P*. kl. *esculentus*, *P*. *lessonae* and *P*. *ridibundus*) and *Rana temporaria* are common species, but the *Pelobates fuscus* (spadefoot toad) is considered ‘threatened’ in the Netherlands [21]. *Pelobates fuscus* was found to be infected at two sites. At one of these sites (no.47), the species had just been reintroduced (Table 2). At this site, there was concurrent ranavirus disease in *Pelophylax* spp., and the virus was phylogenetically distinct from the virus associated with the outbreaks at site no.25. (Table 3; Fig 5).

**Table 3:** Severity and frequency of lesions per species at sites with or without ranavirus.

Ranavirus at site	Host species	Site		No. of sites	No. of speci-mens	Average lesion score (proportion of specimens with lesion)		
		Phylo-group at site	Km to DNP			ICIB	Necrosis	Vascular damage
Yes	*Pelophylax* spp.	Group I	≤ 20	10	27	0.7 (88%)	1.4 (81%)	0.3 (52%)
		Group II	> 150	2	7	0.5 (71%)	0.9 (86%)	0.1 (43%)
	*Lissotriton vulgaris*	Group I	≤ 20	5	9	0.4 (60%)	0.6 (70%)	0.2 (60%)
	*Pelobates fuscus*	Group I	≤ 20	1	11	0.3 (78%)	0.7 (91%)	0.1 (27%)
		Group II	> 150	1	2	0.4 (100%)	0.4 (50%)	0.3 (50%)
	*Bufo bufo*	Group I	≤ 20	1	1	0.4	2.0	0.6
	*Rana temporaria*	Group I	55–60	1	7	0.2 (57%)	0.2 (71%)	0.1 (29%)
	All species	Groups I, II and ND*		18	69	0.5 (75%)	0.9 (77%)	0.2 (43%)
No	*Pelophylax* spp.	NA		10	17	0 (0%)	0.2 (35%)	0.3 (47%)
	*Lissotriton vulgaris*	NA		3	5	0 (0%)	0 (0%)	<0.1(20%)
	*Pelobates fuscus*	NA		1	1	0 (0%)	0 (0%)	0 (0%)
	*Bufo bufo*	NA		9	21	0 (0%)	0.2 (38%)	0.1 (38%)
	*Rana temporaria*	NA		13	40	0.1 (18%)	0.3 (55%)	0.2 (48%)
	*Epidalea calamita*†	NA		1	1	0 (0%)	0 (0%)	0 (0%)
	*Ichthyosaura alpestris*‡	NA		1	1	0 (0%)	0 (0%)	0 (0%)
	All species			34	86	<0.1 (8%)	0.2 (42%)	0.2 (43%)

* Scores in specimens from four sites where the ranavirus group was not determined (ND; 1 *Pelophylax* sp. site no. 38; 2 *Lissotriton vulgaris* from site no. 43; 1 *Pelophylax* sp. site no. 49; 1 *Bufo bufo* from site no.52) were not detailed in the table, but were included in the “all specimens” scores.

† *Epidalea calamita* = natterjack toad

‡ *Ichthyosaura alpestris =* Alpine newt

Group I ranaviruses at sites ≤ 20 km of DNP were associated with mass mortality events involving mainly *Pelophylax* spp. and *Lissotriton vulgaris* (Table 2). Tens to hundreds of (sub-) adults died as well as larval and juvenile stages at each site. Subsequent submissions from a few of these sites and long-term monitoring in DNP [30] confirmed continued ranavirus presence (e.g. site no.25, Fig 5), and *Pelophylax* spp. in particular were either not observed or observed in lower numbers than prior to the outbreak (Table 2).

The presence of ICIB and of necrosis were good indicators for ranavirus infection, but the presence of vascular damage was not (Table 3). Only effects of vascular damage (hemorrhages) may be visible macroscopically and thus be detected by submitters; however, this lesion was not significantly associated with ranavirus infection at a site: there was vascular damage in 30/69 (43%) specimens at sites where ranavirus cases were detected and in 37/86 (43%) specimens at sites where mortality was not ranavirus associated (Yates’*X*^*2*^ = 0.011, df = 1, p = 0.916). In contrast, the lesions that could only be observed histologically (presence of ICIB, and to lesser extent necrosis) were associated with ranavirus infection at sites in this study. The presence of ICIB was significantly more frequent among dead amphibians submitted from sites where ranavirus was found (52/69), than where it was not found (7/86; Yates’*X*^*2*^ = 70.558, df = 1, p = 0). The presence of necrosis was significantly more frequent among dead amphibians submitted from sites where ranavirus was found (53/69), than where it was not found (36/86; Yates’*X*^*2*^ = 17.725, df = 1, p = 0.00003).

At sites where ranavirus was present, there were significant interspecies differences in the number of ICIB (ranavirus assembly sites [31]; ANOVA, F-statistic = 4.69, df = 3, p = 0.005), and the extent of necrosis in tissues (ANOVA, F-statistic = 8.46, df = 3, p = 0.00008), with the most severe lesions observed in *Pelophylax* spp. (Table 3; S4 Text).

### The DNP outbreak is likely to continue to spread

Assuming that the group I ranavirus involved in the outbreak at DNP (site no. 0) had spread to the detected sites in its vicinity where specimens of different life stages, including adults, died in high numbers (i.e., sites no. 7, 9, 11 and 13 in 2011; site no. 25 in 2012, sites no. 35, 36, 37 and 38 in 2013; and sites no. 45, 48 and 51 in 2014), the discrete reproductive power of this outbreak was calculated to be 0.353, and the 95% confidence interval (0; 0.521). In other words, in this outbreak the probability of the disease spreading to another site was estimated as 0.35 (0; 0.52). The probability of no reproduction four years in a row was 0.18. So in 18% of the cases there will be no reproduction after the initial case. In the 1000 Monte Carlo runs this happened 179 times. Considering only the cases where after the initial site at least one other site was infected (about 82% of the cases), i.e., those cases where reproduction occurred, the 95% confidence interval for the discrete reproductive power is (0.143; 0.523).

## Discussion

Through the integration of pathology, epidemiology and molecular biology, this study provides evidence for a CMTV-like epidemic disease outbreak occurring among wild amphibians in the north of the Netherlands. It also highlights differences in severity of ranavirus-induced lesions among affected species, and shows the presence of phylogenetically distinct, geographically segregated groups of ranaviruses in the Dutch wildlife, all of which were characterized as CMTV-like viruses.

The occurrence of an epidemic is substantiated by the expanding temporal-spatial cluster of sites in the vicinity of DNP, where phylogenetically closely related group I ranaviruses were associated with severe lesions and high mortality in amphibians of different life stages. The fact that adult specimens also died in high numbers at these sites, makes it probable that the affected populations lacked innate [32] and protective [33] immunity. Alternative explanations for the mortality in the (sub)-adults, such as high density stress or activity related stress [1] are unlikely, as the (sub-)adults were in a period of low phenological frequency (Table 2). A common environmental stressor [1] is equally implausible, given that the waterbodies were diverse in nature, not interconnected, and similar to waterbodies throughout the country.

It is likely that this group I ranavirus causing the outbreak around DNP will continue to spread to susceptible populations at new sites (discrete reproductive power of the outbreak: 0.35 [95% CI: (0; 0.53)]). Though amphibians generally disperse ≤3 km annually, their home ranges overlap [21], providing opportunity for relay of virus among sites within a season. Other nature-mediated or human-mediated spread may equally occur [1, 7]. Reproductive power can be a useful parameter to evaluate the effectiveness of control measures [22]. In the current study, the reproductive power was kept constant, given that the study period lasted only four years and the surveillance set-up results in a limited number of detections per year. Detection of changes in the reproductive power of this outbreak throughout time can be achieved by continuing surveillance in a similar fashion in the upcoming years.

Among the six common native amphibian species shown to be infected by the detected CMTV-like ranaviruses, *Pelophylax* spp. were most affected in terms of severity of lesions, mortality and sustained local effects on population size. While the biological basis for this apparent susceptibility is at the moment unclear, *Pelophylax* kl. *esculentus* has previously been linked to CMTV-like ranavirus die-offs elsewhere [10, 12, 14].

Besides common amphibian species, *Pelobates fuscus* was shown to be susceptible. This is one of the eight amphibian species considered threatened in the Netherlands [21]. The introduction of a novel multi-host pathogen such as ranavirus may present a local extinction risk for any of the few remaining small populations of these species [34]. Several re-introduction projects have been implemented in the Netherlands since 2000 for *Pelobates fuscus* as well as three other species [35]. Such projects translocate specimens which implicates a risk for disease transmission [36]. In this study, the *Pelobates fuscus* larvae raised from eggs taken from a confirmed ranavirus site (no. 25) were unlikely to have caused the ranavirus associated mortality at the site of their re-introduction (no. 47) for several reasons. First, there were retrospective reports of water frogs dying at the site just before the reintroduction took place. Next, there was no evidence of disease prior to the release of the *Pelobates fuscus* larvae, and the larvae that remained in the captive facility tested negative for ranavirus by PCR. Finally, the ranavirus circulating at the site from which the eggs were taken belonged to a different phylogenetic group. This example reiterates the importance of pre-translocation disease risk assessments in conservation programs [36].

CMTV-like ranaviruses, seemingly associated with lower mortality, were detected in geographical areas non-adjacent to DNP from 2013 onwards, and included ranaviruses from a different phylogenetic group. It is possible that CMTV-like ranaviruses are being widely introduced through a, perhaps trade-related, pathway [37–38], with varying success in becoming established. Alternatively, CMTV-like ranaviruses may be endemic to the Netherlands, or even Europe [5], and the strain emerging around DNP may be novel to the populations of the area, possibly indicating a lack of co-evolution [39]. Both scenarios are compatible with the fact that the detected ranaviruses also cluster closely with ADRV which to the best of our knowledge has not been detected so far in free-living wild specimens [27–29]. Multidisciplinary coordinated research at international level may clarify this matter.

## Supporting Information

S1 Text (PDF)

S2 Text (PDF)

S3 Text (PDF)

S4 Text (PDF)

S1 Table (PDF)

S2 Table (PDF)

S3 Table (PDF)

S4 Table (PDF)
