# Supplementary material for: Investigation of Amphibian Mortality Events in Wildlife Reveals an On-Going Ranavirus Epidemic in the North of the Netherlands
Source: PLoS One. 2016 Jun 17;11(6):e0157473. doi: 10.1371/journal.pone.0157473 (PMC4912076; doi:10.1371/journal.pone.0157473)
Supplement: S1 Text — (PDF) [file pone.0157473.s001.pdf]

S1 Text

Immunohistochemistry method

For immunohistochemical staining, a polyclonal rabbit anti-European catfish virus antibody (kindly donated by G. Bovo, Instituto Zooprofilattico Sperimentale delle Venezie, Italy) was used as a primary antibody. Tissues were fixed in 4% phosphate buffered formalin for 24 hours, and paraffin embedded. Subsequently, the tissues were dewaxed and hydrated through decreasing concentrations of alcohol, and gently washed twice with Tris buffered saline (TBS, pH 7.2) for 5 minutes. Endogenous peroxidase activity was inhibited by immersion of the slides in a 4% hydrogen peroxide for 30 minutes. Blocking was achieved by placing the slides on TBS buffer with 5% bovine serum albumin (BSA) for 30 min at room temperature. Afterwards, slides were incubated with a polyclonal rabbit anti-European catfish virus antibody diluted to 1:1800 in PBS buffer with 2.5% BSA for 1 hour at room temperature. After washing three times with TBS-Tween 0.1%, a biotinylated goat anti-rabbit IgG antibody (Dako) diluted to 1:250 in TBS buffer with 2.5% BSA was added, and followed by a 30 minute incubation period. The slides were then washed with TBS-Tween three times, incubated with Avidin-Biotin Complex (Dako) diluted to 1:250 in PBS buffer for 30 minutes at room temperature, washed with PBS for 5 minutes, and a chromogen substrate was added (Fast Red, Kem-En-Tec) for 8 minutes. Afterwards, the slides were placed in running tap water for 5 minutes, counterstained with hematoxylin for one minute. Finally, after a 10 minute bath in running tap water, the slides were mounted with Aquamount (Merck), and examined under a light microscope. Negative controls included tissues from non ranavirus infected amphibians, tissues omitting primary or secondary antibody and an isotype control.
