# Supplementary material for: Investigation of Amphibian Mortality Events in Wildlife Reveals an On-Going Ranavirus Epidemic in the North of the Netherlands
Source: PLoS One. 2016 Jun 17;11(6):e0157473. doi: 10.1371/journal.pone.0157473 (PMC4912076; doi:10.1371/journal.pone.0157473)
Supplement: S2 Text — (PDF) [file pone.0157473.s002.pdf]

S2 Text

Model script details

```
inf <- c(1,4,1,4,3) #dataset

################################################################################

library(stats4)

library(bbmle)

# cumulative number of inf:

c.inf <- cumsum(inf)

#

ynu <- c.inf[-1]

yprev <- c.inf[-length(inf)]

t <- 1:(length(inf)-1)

#delt=t-c(0,t[-length(t)])

################################################################################

pexp <- function(q,l,lower.tail=TRUE){

  if (any(q <= 0))

    stop("q must contain positive values")

  if (any(l <= 0))

    stop("l must be positive")

  prob=1-exp(-l*q)

  if(!lower.tail) prob <- 1-prob

  prob

}

################################################################################

# the likelihood function and the exponential distribution

#
```

```
min.loglik.ex <- function(ll,tijd,yt,yp) {
  #
  rep.pow <- c(1-pexp(1,exp(ll),lower.tail=FALSE),1-pexp(tijd[-
1],exp(ll),lower.tail=FALSE)/pexp(tijd[-length(tijd)],exp(ll),lower.tail=FALSE))
  -sum(dnbinom(yt-yp,size=yp,prob=1-rep.pow,log=TRUE))
}
#
####################################################################
# Fit t model with neg. binomial and constant rep. power
fit.exp <- mle2(min.loglik.ex,start=list(ll=-
.5),data=list(tijd=t,yt=ynu,yp=yprev),control=list(trace=0,type=1,REPORT=1,maxit=50000,reltol=10^
(10)*.Machine$double.eps),method="Nelder-Mead")
#
summary(fit.exp)

# discrete rep.power
1-exp(-exp(coef(fit.exp)))
logLik(fit.exp)
AIC(fit.exp)
vcov(fit.exp)
prof <- profile(fit.exp,which=1)
plot(prof)

####################################################################
# the parametric bootstrap
rep.h1=NULL
#rep.h2=NULL
for (k in 1:1000){
```

```
  y <- NULL
  y[1]=1
  for (j in 2:5){
       y[j] <- y[j-1]+rnbinom(1,size=y[j-1], prob=exp(log(1-(1-exp(-exp(coef(fit.exp)))))))
  }
  yprev=y[-length(y)];ynu=y[-1];t=1:4
  fit <- mle2(min.loglik.ex,start=list(ll=-
.5),data=list(tijd=t,yt=ynu,yp=yprev),control=list(trace=0,type=1,REPORT=1,maxit=50000,reltol=10^
(10)*.Machine$double.eps),method="Nelder-Mead")
  rep.h1[k]=1-exp(-exp(coef(fit)))
#  rep.h2[k]=1-sum(yprev)/sum(ynu) #ml-estimator
  lines(0:4,y,type="s",col="grey")
}
#
quantile(rep.h1,probs=c(.025,.975))
#
hist(rep.h1)
#
# confidence interval considering only those cases where there was reproduction
plot(c(0,4),c(0,50),pch=" ",xlab="Time",ylab="Cumm.no. cases")
lines(0:4,c.inf,type="s",col="black",lwd=2)
reph.pos=rep.h1[rep.h1>0]
table(rep.h1>0)
quantile(reph.pos,probs=c(.025,.975))
```
