# Supplementary material for: Investigation of Amphibian Mortality Events in Wildlife Reveals an On-Going Ranavirus Epidemic in the North of the Netherlands
Source: PLoS One. 2016 Jun 17;11(6):e0157473. doi: 10.1371/journal.pone.0157473 (PMC4912076; doi:10.1371/journal.pone.0157473)
Supplement: S3 Text — (PDF) [file pone.0157473.s003.pdf]

S3 Text

Other pathogens and lesions

In specimens from amphibian mortality events associated with ranavirus, lesions/infections not directly related to ranavirus infection were rare, and usually not severe. These included parasites in gastro-intestinal tract in specimens from several sites; mild gastritis in one *Pelophylax* kl. *esculentus* from site no. 30; and protozoa in the skin of a *Lissotriton vulgaris* from site no. 36. The three *Pelophylax* spp. larvae from site no. 49 had edematous viscera in the coelomic cavity, but no other lesions. The single specimen from site no. 52 was a *Bufo bufo* that had pneumonia and nematodes in the lungs. Bacterial or fungal overgrowth or both, i.e., post-mortem contamination, occurred in specimen at 6/18 sites. When suspected, PCR-tests were performed by Gent University to check for presence of *Batrachochytrium dendrobatis* genetic material (chytridiomycosis), as detailed in reference number 3 of the manuscript. Test results were always negative.
