# Supplementary material for: Investigation of Amphibian Mortality Events in Wildlife Reveals an On-Going Ranavirus Epidemic in the North of the Netherlands
Source: PLoS One. 2016 Jun 17;11(6):e0157473. doi: 10.1371/journal.pone.0157473 (PMC4912076; doi:10.1371/journal.pone.0157473)
Supplement: S4 Text — (PDF) [file pone.0157473.s004.pdf]

S4 Text

Interspecies differences in lesions

The ranavirus-associated lesions examined for interspecies differences were the numbers of ICIB and extent of necrosis; “n” indicates the total number of specimens scored per species, obtained from sites with confirmed ranavirus associated mortality events.

There were significant species differences (ANOVA, F-statistic = 4.69, df = 3, p = 0.005) in numbers of ICIB among *Pelophylax* spp. (0.67, 95%CI [0.65;0.70]; n = 36), *Pelobates fuscus* (0.35, 95%CI [0.30;0.39]; n = 13), *Lissotriton vulgaris* (0.40, 95%CI [0.33;0.46]; n = 11) and *Rana temporaria* (0.22, 95%CI [0.15; 0.30]; n = 7) from sites with CMTV-like ranavirus mortality events.

There were also significant species differences (ANOVA, F-statistic = 8.46, df = 3, p = 0.00008) in extent of necrosis among *Pelophylax* spp. (1.24, 95%CI [1.20;1.28]; n = 36), *Pelobates fuscus* (0.64, 95%CI [0.57;0.71]; n = 13), *Lissotriton vulgaris* (0.47, 95%CI [0.38;0.56]; n = 11) and *Rana temporaria* (0.18, 95%CI [0.14;0.22]; n = 7) from sites with CMTV-like ranavirus mortality events. *Bufo bufo* (n = 2) were excluded due to their low numbers.
