# Supplementary material for: Investigation of Amphibian Mortality Events in Wildlife Reveals an On-Going Ranavirus Epidemic in the North of the Netherlands
Source: PLoS One. 2016 Jun 17;11(6):e0157473. doi: 10.1371/journal.pone.0157473 (PMC4912076; doi:10.1371/journal.pone.0157473)
Supplement: S1 Table — (PDF) [file pone.0157473.s005.pdf]

S1 Table

Complete ranavirus genomes obtained from GenBank

Full name of the virus isolate	GenBank accesion number	Host species isolated from	Country	Depicted in the phylogenetic tree as
Common midwife toad ranavirus isolate Pelophylax kl.esculentus/2013/NL, complete genome	KP056312	*Pelophylax kl.esculentus*	The Netherlands	CMTV KP056312 P.kl esculentus
Common midwife toad ranavirus isolate Mesotriton alpestris/2008/E, complete genome	JQ231222	*Mesotriton alpestris*	Spain	CMTV JQ231222 M.alpestris
Frog virus 3, complete genome	AY548484	*Lithobates pipiens*	USA	FV3 AY548484 L pipiens
Frog virus 3 isolate SSME, complete genome	KJ175144	*Lithobates pipiens*	USA	FV3SMME KJ175144 L.pipiens
Tortoise ranavirus isolate 1 (882/96), complete genome.	KP266743	*Testudo kleinmanni*	Germany	TRV KP 266743 T.kleimanni
Testudo hermanni ranavirus isolate CH8/96, complete genome	KP266741	*Testudo hermanni*	Germany	THRV KP266741 T hermmani
German gecko ranavirus isolate 2000/99, complete genome	KP266742	*Uroplatus fimbriatus*	Germany	GGRV KP266742 U.fimbriatus
Tiger frog virus, complete genome	AF389451	*Rana tigrina*	China	TFV AF349451 R.tigrina
Soft shelled turtle iridovirus, complete genome	EU627010	*Trionix sinensis*	China	STIV EU627010 T.sinensis
European sheathfish virus, complete genome	JQ724856	*Silurus glanis*	Spain	ESV JQ724856 S.glanis
Epizootic hematopoietic necrosis virus, complete genome	FJ433873	*Perca fluviatis*	Australia	EHNV FJ433873 P.fluviatis
Ambystoma tigrinum stebbensi virus, complete genome	AY150217	*Ambystoma tigrinum stebbensi*	USA	ATV AY150217 A. tigrinum
Andrias davidianus ranavirus isolate 1201, complete genome	KC865735	*Andrias davidianus*	China	ADRV KC865735 A.davidianus
Andrias davidianus r anavirus ranavirus 2010SX,complete genome	KF033124	*Andrias davidianus*	China	ADRV KF033124 A.davidianus
Chinese Giant Salamander Iridovirus, isolate CGSIV-HN1104, complete geonme	KF512820	*Andrias davidianus*	China	CGSI KF512820 A.davidianus
Rana grylio ranavirus, complete genome	JQ654586	*Rana grylio*	China	RGV JQ654586 R.grylio
Singapore grouper iridovirus, complete genome	AY521625	*Epinephelus awoara*	Singapore	Not depicted
Grouper Iridovirus, complete genome	AY666015	*Epinephelus awoara*	China	Not depicted
