# Supplementary material for: Investigation of Amphibian Mortality Events in Wildlife Reveals an On-Going Ranavirus Epidemic in the North of the Netherlands
Source: PLoS One. 2016 Jun 17;11(6):e0157473. doi: 10.1371/journal.pone.0157473 (PMC4912076; doi:10.1371/journal.pone.0157473)
Supplement: S2 Table — (PDF) [file pone.0157473.s006.pdf]

# S2 Table

# Partial ranavirus gene sequences obtained from GenBank

| Full name of the virus isolate | GenBank accesion number | Host species it was isolated from | Country | Depicted in the phylogenetic tree as |
|---|---|---|---|---|
| Bosca's newt virus isolate GA11002 eukaryote initiation factor gene, partial cds | KJ703114 | *Lissotriton boscai* | Spain | BNV GA11002 L.boscai |
| Common midwife toad ranavirus isolate PE11001 eukaryote initiation factor gene, partial cds | KJ703115 | *Alytes obstetricans* | Spain | CMTV PE11001 A.obstetricans |
| Common midwife toad ranavirus isolate PE11004 eukaryote initiation factor gene, partial cds | KJ703116 | *Rana temporaria* | Spain | CMTV PE11004 R.temporaria |
| Common midwife toad ranavirus isolate PE11114 eukaryte initiation factor gene, partial cds | KJ703117 | *Mesotriton alpestris* | Spain | CMTV PE11114 M.alpestris |
| Bosca's newt virus isolate GA11001 eukaryote initiation factor gene, partial cds | KJ703118 | *Natrix maura* | Spain | BNV GA11001 N.maura |
| Bosca's newt virus isolate GA11010 eukaryote initiation factor gene, partial cds | KJ703119 | *Triturus marmoratus* | Spain | BNV GA11010 T.marmoratus |
| Bosca's newt virus isolate GA11002 major capsid portein gene, partial cds | KJ703120 | *Lissotriton boscai* | Spain | BNV GA11002 L.boscai |
| Bosca's newt virus isolate GA11010 major capsid protein gene, partial cds | KJ703121 | *Triturus marmoratus* | Spain | BNV GA11010 T.marmoratus |
| Bosca's newt virus isolate GA11001 major capsid protein gene, partial cds | KJ703122 | *Natrix maura* | Spain | BNV GA11001 N.maura |
| Common midwife toad ranavirus isolate PE11001 major capsid protein gene, partial cds | KJ703124 | *Alytes obstetricans* | Spain | CMTV PE11001 A.obstetricans |
| Common midwife toad ranavirus isolate PE11114 major capsid protein gene,partial cds | KJ703125 | *Mesotriton alpestris* | Spain | CMTV PE11114 M.alpestris |
| Common midwife toad ranavirus isolate PE11004 major capsid protein gene, partial cds | KJ703126 | *Rana temporaria* | Spain | CMTV PE11004 R.temporaria |
| Bosca's newt virus isolate GA11001 hypothetical protein gene, partial cds | KJ703129 | *Natrix maura* | Spain | BNV GA11001 N.maura |
| Bosca's newt virus isolate GA11002 hypothetical protein gene, partial cds | KJ703130 | *Lissotriton boscai* | Spain | BNV GA11002 L.boscai |
| Bosca's newt virus isolate GA11010 hypothetical protein gene, partial cds | KJ703131 | *Triturus marmoratus* | Spain | BNV GA11010 T.marmoratus |
| Common midwife toad ranavirus isolate PE11004 hypothetical protein gene, partial cds | KJ703133 | *Rana temporaria* | Spain | CMTV PE11004 R.temporaria |
| Common midwife toad ranavirus isolate PE11001 hypothetical protein gene, partial cds | KJ703134 | *Alytes obstetricans* | Spain | CMTV PE11001 A.obstetricans |
| Common midwife toad ranavirus isolate PE11114 hypothetical protein gene, partial cds | KJ703135 | *Mesotriton alpestris* | Spain | CMTV PE11114 M.alpestris |
| Bosca's newt virus isolate GA11001 hypothetical protein gene, partial cds | KJ703137 | *Natrix maura* | Spain | BNV GA11001 N.maura |
| Bosca's newt virus isolate GA11002 hypothetical protein gene, partial cds | KJ703138 | *Lissotriton boscai* | Spain | BNV GA11002 L.boscai |
| Bosca's newt virus isolate GA11010 hypothetical protein gene partial cds | KJ703139 | *Triturus marmoratus* | Spain | BNV GA11010 T.marmoratus |
| Common midwife toad ranavirus isolate PE11004 hypothetical protein gene, partial cds | KJ703140 | *Rana temporaria* | Spain | CMTV PE11004 R.temporaria |
| Common midwife toad ranavirus isolate PE11114 hypothetical protein gene, partial cds | KJ703141 | *Mesotriton alpestris* | Spain | CMTV PE11114 M.alpestris |
| Common midwife toad ranavirus isolate PE11001 hypothetical protein gene, partial cds | KJ703142 | *Alytes obstetricans* | Spain | CMTV PE11001 A.obstetricans |
| Bosca's newt virus isolate GA11010 hypothetical protein gene partial cds | KJ703143 | *Triturus marmoratus* | Spain | BNV GA11010 T.marmoratus |
| Bosca's newt virus isolate GA11002 hypothetical protein gene, partial cds | KJ703144 | *Lissotriton boscai* | Spain | BNV GA11002 L.boscai |
| Bosca's newt virus isolate GA11001 hypothetical protein gene, partial cds | KJ703145 | *Natrix maura* | Spain | BNV GA11001 N.maura |
| Common midwife toad ranavirus isolate PE11114 hypothetical protein gene, partial cds | KJ703147 | *Mesotriton alpestris* | Spain | CMTV PE11114 M.alpestris |
| Common midwife toad ranavirus isolate PE11001 hypothetical protein gene,partial cds | KJ703148 | *Alytes obstetricans* | Spain | CMTV PE11001 A.obstetricans |
| Common midwife toad ranavirus isolate PE11004 hypothetical protein gene, partial cds | KJ703149 | *Rana temporaria* | Spain | CMTV PE11004 R.temporaria |
| Common midwife toad ranavirus isolate PE11004 proliferating cell nuclear antigen gene, partial cds | KJ703150 | *Rana temporaria* | Spain | CMTV PE11004 R.temporaria |
| Common midwife toad ranavirus isolate PE11001 proliferating cell nuclear antigen gene, partial cds | KJ703151 | *Alytes obstetricans* | Spain | CMTV PE11001 A.obstetricans |
| Common midwife toad ranavirus isolate PE11114 proliferating cell nuclear antigen gene,partial cds | KJ703152 | *Mesotriton alpestris* | Spain | CMTV PE11114 M.alpestris |
| Bosca's newt virus isolate GA11001 proliferating cell nuclear antigen gene, partial cds | KJ703154 | *Natrix maura* | Spain | BNV GA11001 N.maura |
| Bosca's newt virus isolate GA11002 proliferating cell nuclear antigen gene, partial cds | KJ703155 | *Lissotriton boscai* | Spain | BNV GA11002 L.boscai |
| Common midwife toad ranavirus isolate PE11004 p31k gene, partial cds | KJ703157 | *Rana temporaria* | Spain | CMTV PE11004 R.temporaria |
| Common midwife toad ranavirus isolate PE11001 p31k gene, partial cds | KJ703158 | *Alytes obstetricans* | Spain | CMTV PE11001 A.obstetricans |
| Common midwife toad ranavirus isolate PE11114 p31k gene, partial cds | KJ703160 | *Mesotriton alpestris* | Spain | CMTV PE11114 M.alpestris |
| Bosca's newt virus isolate GA11002 p31k gene, partial cds | KJ703161 | *Lissotriton boscai* | Spain | BNV GA11002 L.boscai |
| Bosca's newt virus isolate GA11010 p31k gene, partial cds | KJ703162 | *Triturus marmoratus* | Spain | BNV GA11010 T.marmoratus |
| Bosca's newt virus isolate GA11001 p31k gene, partial cds | KJ703163 | *Natrix maura* | Spain | BNV GA11001 N.maura |
