# Supplementary material for: Investigation of Amphibian Mortality Events in Wildlife Reveals an On-Going Ranavirus Epidemic in the North of the Netherlands
Source: PLoS One. 2016 Jun 17;11(6):e0157473. doi: 10.1371/journal.pone.0157473 (PMC4912076; doi:10.1371/journal.pone.0157473)
Supplement: S3 Table — (PDF) [file pone.0157473.s007.pdf]

S3 Table

Partial gene sequence comparison of Dutch ranaviruses to Common midwife toad virus and Andrias davidianus ranavirus

The identity of the partial MCP gene of the ranaviruses detected at the sites in comparison to a common midwife toad virus from Spain and *Andrias davidianus* ranavirus in GenBank.

Distance from index site (km)	Site number	Year	MCP ORF16 (length 462 bp)		HPG ORF13 (length 292 bp)	
			**CMTV 2008, accession number JQ231222.1**	**ADRV 2010, accession number KC865735.1**	**CMTV 2008, accession number JQ231222.1**	**ADRV 2010, accession number KC865735.1**
NA	0	2010	100%	99.78% (1 nucleotide difference)	97.26% (8 nucleotide differences)	97.6% (7 nucleotide differences)
≤ 20 km NPD	7	2011	100%	99.78% (1 nucleotide difference)	97.26% (8 nucleotide differences)	97.6% (7 nucleotide differences)
	9	2011	100%	99.78% (1 nucleotide difference)	97.26% (8 nucleotide differences)	97.6% (7 nucleotide differences)
	11	2011	100%	99.78% (1 nucleotide difference)	97.26% (8 nucleotide differences)	97.6% (7 nucleotide differences)
	13	2011	100%	99.78% (1 nucleotide difference)	97.26% (8 nucleotide differences)	97.6% (7 nucleotide differences)
	25*	2012	100%	99.78% (1 nucleotide difference)	97.26% (8 nucleotide differences)	97.6% (7 nucleotide differences)
	25†	2014	100%	99.78% (1 nucleotide difference)	97.26% (8 nucleotide differences)	97.6% (7 nucleotide differences)
	35	2013	100%	99.78% (1 nucleotide difference)	97.26% (8 nucleotide differences)	97.6% (7 nucleotide differences)
	36	2013	100%	99.78% (1 nucleotide difference)	97.26% (8 nucleotide differences)	97.6% (7 nucleotide differences)
	37‡	2013	100%	99.78% (1 nucleotide difference)	97.26% (8 nucleotide differences)	97.6% (7 nucleotide differences)
	37†	2013	100%	99.78% (1 nucleotide difference)	97.26% (8 nucleotide differences)	97.6% (7 nucleotide differences)
	38	2013	100%	99.78% (1 nucleotide difference)	Not available	Not available
	45	2014	100%	99.78% (1 nucleotide difference)	97.26% (8 nucleotide differences)	97.6% (7 nucleotide differences)
	48	2014	100%	99.78% (1 nucleotide difference)	97.26% (8 nucleotide differences)	97.6% (7 nucleotide differences)
	51	2014	100%	99.78% (1 nucleotide difference)	97.26% (8 nucleotide differences)	97.6% (7 nucleotide differences)
55-60 km	30	2013	100%	99.78% (1 nucleotide difference)	96.91% (9 nucleotide differences)	97.26% (8 nucleotide differences)
	43	2014	100%	99.78% (1 nucleotide difference)	Not available	Not available
	52	2014	100%	99.78% (1 nucleotide difference)	Not available	Not available
>150 km	33	2013	99.78% (1 nucleotide difference)	99.56% (2 nucleotides difference)	97.26% (8 nucleotide differences)	98.97% (3 nucleotide differences)
	47	2014	99.35% (3 nucleotide differences)	99.56% (2 nucleotide differences)	97.26% (8 nucleotide differences)	98.97% (3 nucleotide differences)
	49	2014	100%	99.56% (2 nucleotide differences)	Not available	Not available

* *Pelobates fuscus (Pf)*
† *Lissotriton vulgaris (Lv)*
‡ *Pelophylax* spp. (P).

PCNA ORF 22 (length 317 bp)		HPG ORF 58(length 326 bp)		HPG ORF 59 (
CMTV 2008, accession number JQ231222.1	ADRV 2010, accession number KC865735.1	CMTV 2008, accession number JQ231222.1	ADRV 2010, accession number KC865735.1	CMTV 2008, accession number JQ231222.1
99.68% (1 nucleotide difference)	99.68% (1 nucleotide difference)	98.77% (4 nucleotide differences)	99.38% (2 nucleotide differences)	99.33% (2 nucleotide differences)
99.68% (1 nucleotide difference)	99.68% (1 nucleotide difference)	99.38% (2 nucleotide differences)	100%	99% (3 nucleotide differences)
99.68% (1 nucleotide difference)	99.68% (1 nucleotide difference)	99.38% (2 nucleotide differences)	100%	99.33% (2 nucleotide differences)
99.68% (1 nucleotide difference)	99.68% (1 nucleotide difference)	99.38% (2 nucleotide differences)	100%	99.33% (2 nucleotide differences)
99.68% (1 nucleotide difference)	99.68% (1 nucleotide difference)	99.38% (2 nucleotide differences)	100%	99.33% (2 nucleotide differences)
99% (3 nucleotide differences)	99% (3 nucleotide differences)	99.38% (2 nucleotide differences)	100%	99% (3 nucleotide differences)
99.68% (1 nucleotide difference)	99.68% (1 nucleotide difference)	99.38% (2 nucleotide differences)	100%	99.33% (2 nucleotide differences)
99.68% (1 nucleotide difference)	99.68% (1 nucleotide difference)	99.38% (2 nucleotide differences)	100%	99.33% (2 nucleotide differences)
99.68% (1 nucleotide difference)	99.68% (1 nucleotide difference)	99.38% (2 nucleotide differences)	100%	99.33% (2 nucleotide differences)
99.68% (1 nucleotide difference)	99.68% (1 nucleotide difference)	99.38% (2 nucleotide differences)	100%	99.33% (2 nucleotide differences)
99.68% (1 nucleotide difference)	99.68% (1 nucleotide difference)	99.38% (2 nucleotide differences)	100%	99.33% (2 nucleotide differences)
Not available	Not available	Not available	Not available	Not available
99.68% (1 nucleotide difference)	99.68% (1 nucleotide difference)	99.38% (2 nucleotide differences)	100%	99.33% (2 nucleotide differences)
99.68% (1 nucleotide difference)	99.68% (1 nucleotide difference)	99.38% (2 nucleotide differences)	100%	99.33% (2 nucleotide differences)
99.68% (1 nucleotide difference)	99.68% (1 nucleotide difference)	99.38% (2 nucleotide differences)	100%	99.33% (2 nucleotide differences)
99.68% (1 nucleotide difference)	99.68% (1 nucleotide difference)	99% (3 nucleotide differences)	99.69% (1 nucleotide difference)	99% (3 nucleotide differences)
Not available	Not available	Not available	Not available	Not available
Not available	Not available	Not available	Not available	Not available
99.68% (1 nucleotide difference)	99.68% (1 nucleotide difference)	98.77% (4 nucleotide differences)	99.38% (2 nucleotide differences)	99.33% (2 nucleotide differences)
99.68% (1 nucleotide difference)	99.68% (1 nucleotide difference)	98.77% (4 nucleotide differences)	99.38% (2 nucleotide differences)	99.33% (2 nucleotide differences)
Not available	Not available	Not available	Not available	Not available

(length 300 bp)	EIF2alfa ORF 81 (length 345 bp)		p31k ORF 82 (325 bp)	
**ADRV 2010, accession number KC865735.1**	**CMTV 2008, accession number JQ231222.1**	**ADRV 2010, accession number KC865735.1**	**CMTV 2008, accession number JQ231222.1**	**ADRV 2010, accession number KC865735.1**
99.33% (2 nucleotide differences)	97.97% (7 nucleotide differences)	98.55% (5 nucleotide differences)	99.69% (1 nucleotide difference)	100%
99% (3 nucleotide differences)	98.26% (6 nucleotide differences)	98.84% (4 nucleotide differences)	99.69% (1 nucleotide difference)	100%
99.33% (2 nucleotide differences)	98.26% (6 nucleotide differences)	98.84% (4 nucleotide differences)	99.69% (1 nucleotide difference)	100%
99.33% (2 nucleotide differences)	98.26% (6 nucleotide differences)	98.84% (4 nucleotide differences)	99.69% (1 nucleotide difference)	100%
99.33% (2 nucleotide differences)	98.26% (6 nucleotide differences)	98.84% (4 nucleotide differences)	99.69% (1 nucleotide difference)	100%
99% (3 nucleotide differences)	98.26% (6 nucleotide differences)	98.84% (4 nucleotide differences)	99.69% (1 nucleotide difference)	100%
99.33% (2 nucleotide differences)	98.26% (6 nucleotide differences)	98.84% (4 nucleotide differences)	99.69% (1 nucleotide difference)	100%
99.33% (2 nucleotide differences)	98.26% (6 nucleotide differences)	98.84% (4 nucleotide differences)	99.69% (1 nucleotide difference)	100%
99.33% (2 nucleotide differences)	98.26% (6 nucleotide differences)	98.84% (4 nucleotide differences)	99.69% (1 nucleotide difference)	100%
99.33% (2 nucleotide differences)	97.97% (7 nucleotide differences)	98.55% (5 nucleotide differences)	99.69% (1 nucleotide difference)	100%
99.33% (2 nucleotide differences)	98.26% (6 nucleotide differences)	98.84% (4 nucleotide differences)	99.38% (2 nucleotide differences)	99.69% (1 nucleotide difference)
Not available	Not available	Not available	Not available	Not available
99.33% (2 nucleotide differences)	98.26% (6 nucleotide differences)	98.84% (4 nucleotide differences)	99.69% (1 nucleotide difference)	100%
99.33% (2 nucleotide differences)	98.26% (6 nucleotide differences)	98.84% (4 nucleotide differences)	99.69% (1 nucleotide difference)	100%
99.33% (2 nucleotide differences)	98.26% (6 nucleotide differences)	98.84% (4 nucleotide differences)	99.69% (1 nucleotide difference)	100%
99% (3 nucleotide differences)	97.68% (8 nucleotide differences)	98.26% (6 nucleotide differences)	99.38% (2 nucleotide differences)	99.69% (1 nucleotide difference)
Not available	Not available	Not available	Not available	Not available
Not available	Not available	Not available	Not available	Not available
99.33% (2 nucleotide differences)	99.13% (3 nucleotide differences)	99.71% (1 nucleotide difference)	99.69% (1 nucleotide difference)	100%
99.33% (2 nucleotide differences)	98.26% (6 nucleotide differences)	98.84% (4 nucleotide differences)	99.69% (1 nucleotide difference)	100%
Not available	Not available	Not available	Not available	Not available
