# Supplementary material for: Investigation of Amphibian Mortality Events in Wildlife Reveals an On-Going Ranavirus Epidemic in the North of the Netherlands
Source: PLoS One. 2016 Jun 17;11(6):e0157473. doi: 10.1371/journal.pone.0157473 (PMC4912076; doi:10.1371/journal.pone.0157473)
Supplement: S4 Table — (PDF) [file pone.0157473.s008.pdf]

S4 Table

GenBank accession numbers

The accession numbers are given per gene and per virus**.**

Virus (site no.)	GenBank accession no.						
	HP 13R	MCP 16L	PCNA 22L	HP 58L	HP 59R	EIF 81L	P31K 82L
UU3100917003 (no.0)	KT003402	KT003477	KT003447	KT003432	KT003417	KT003492	KT003462
UU3110504006 (no.7)	KT003403	KT003478	KT003448	KT003433	KT003418	KT003493	KT003463
UU3110719031 (no.9)	KT003404	KT003479	KT003449	KT003434	KT003419	KT003494	KT003464
UU3110081001 (no.11)	KT428586	KT428571	KT428574	KT428577	KT428580	KT441016	KT428583
UU3110920007 (no.13)	KT428587	KT428572	KT428575	KT428578	KT428581	KT441017	KT428584
UU3120627007 (no.25)	KT003405	KT003480	KT003450	KT003435	KT003420	KT003495	KT003465
UU3141021029 (no.25)	KT003414	KT003489	KT003459	KT003444	KT003429	KT003502	KT003474
UU3130415034 (no.30)	KT003406	KT003481	KT003451	KT003436	KT003421	KT003496	KT003466
UU3131017037 (no.33)	KT003410	KT003485	KT003455	KT003440	KT003425	KT003499	KT003470
UU3130822030 (no.36)	KT003407	KT003482	KT003452	KT003437	KT003422	KT003497	KT003467
UU3130829030 (no.37)	KT003408	KT003483	KT003453	KT003438	KT003423	KT003498	KT003468
UU3130829033 (no.37)	KT003409	KT003484	KT003454	KT003439	KT003424	KT003505	KT003469
UU3140528027 (no.45)	KT428588	KT428573	KT428576	KT428579	KT428582	KT441018	KT428585
UU3140701037 (no.47)	KT003411	KT003486	KT003456	KT003441	KT003426	KT003506	KT003471
UU3140708068 (no.48)	KT003412	KT003487	KT003457	KT003442	KT003427	KT003500	KT003472
UU3140911033 (no.51)	KT003413	KT003488	KT003458	KT003443	KT003428	KT003501	KT003473
CVI13011489-1 (no.35)	This isolate was fully sequenced. The GenBank accession number for the full genome is: KP056312						
